# Is Platelet-Activating Factor the Missing Link Between COVID-19 and Atherosclerosis?

**DOI:** 10.1016/j.jacadv.2024.101397

**Published:** 2024-11-09

**Authors:** Constantinos A. Demopoulos, Smaragdi Antonopoulou, Theoharis C. Theoharides

**Affiliations:** a National and Kapodistrian University of Athens, Athens, Greece; b School of Health Science and Education, Harokopio University, Athens, Greece; c Institute for Neuro-Immune Medicine, Nova Southeastern University, Clearwater, Florida, USA

We read with interest the recent review by Chidambaram et al.^1^ However, we need to point out the lack of reference to the role of platelet-activating factor (PAF) as the missing link between COVID-19 and atherosclerosis. PAF is the most potent lipid inflammatory factor,^2^ which has gained attention for its dual role in COVID-19 and atherosclerosis.^3^

PAF is known to play a pivotal role in the pathogenesis of atherosclerosis by promoting endothelial dysfunction, inflammation, and platelet aggregation.^4^ Interestingly, SARS-CoV-2 induces a hyperinflammatory state, often termed a "cytokine storm," which can exacerbate pre-existing atherosclerotic conditions or initiate new vascular inflammatory events that can lead to or exacerbate cardiovascular symptomatology.

PAF is implicated in inflammation which can trigger a local vascular reaction within arterial plaques, resulting in thrombosis, plaque disruption, and acute ischemic events.^2^ Endothelial cell dysfunction, an early event, and the subsequent platelet activation and adhesion to the activated endothelium are central to both atherosclerosis and COVID-19. Moreover, SARS-CoV-2 spike protein can stimulate the release of PAF from monocytes, triggering cytokine release and perivascular inflammation.^5^

Understanding the role of PAF in COVID-19 could open new therapeutic avenues. Targeting PAF signaling pathways may not only mitigate the severe inflammatory responses associated with the virus but also reduce the cardiovascular complications that contribute significantly to the morbidity and mortality of COVID-19 patients. Recognizing the role of PAF in this context can enhance our approach to treatment and potentially improve outcomes for patients affected by both COVID-19 and atherosclerosis.
